# Redefining the scientific method: As the use of sophisticated scientific methods that extend our mind

**DOI:** 10.1093/pnasnexus/pgae112

**Published:** 2024-03-12

**Authors:** Alexander Krauss

**Affiliations:** London School of Economics, London, UK; Institute for Economic Analysis, Spanish National Research Council, Barcelona, Spain

**Keywords:** scientific method, scientific methodology, scientific discovery, scientific progress

## Abstract

Scientific, medical, and technological knowledge has transformed our world, but we still poorly understand the nature of scientific methodology. Science textbooks, science dictionaries, and science institutions often state that scientists follow, and should follow, the universal scientific method of testing hypotheses using observation and experimentation. Yet, scientific methodology has not been systematically analyzed using large-scale data and scientific methods themselves as it is viewed as not easily amenable to scientific study. Using data on all major discoveries across science including all Nobel Prize and major non-Nobel Prize discoveries, we can address the question of the extent to which “the scientific method” is actually applied in making science's groundbreaking research and whether we need to expand this central concept of science. This study reveals that 25% of all discoveries since 1900 did not apply the common scientific method (all three features)—with 6% of discoveries using no observation, 23% using no experimentation, and 17% not testing a hypothesis. Empirical evidence thus challenges the common view of the scientific method. Adhering to it as a guiding principle would constrain us in developing many new scientific ideas and breakthroughs. Instead, assessing all major discoveries, we identify here a general, common feature that the method of science can be reduced to: making all major discoveries has required using sophisticated methods and instruments of science. These include statistical methods, particle accelerators, and X-ray methods. Such methods extend our mind and generally make observing, experimenting, and testing hypotheses in science possible, doing so in new ways and ensure their replicability. This provides a new perspective to the scientific method—embedded in our sophisticated methods and instruments—and suggests that we need to reform and extend the way we view the scientific method and discovery process.

Science is fascinating because discoveries like new vaccines, more efficient forms of electricity generation, and new medical therapies can spread across the globe and improve the lives of many people. Science and discoveries have enhanced our ability to understand and predict many aspects of our natural and social world. Einstein's special relativity revolutionized physics in the 20th century and how we understand the relationship between space and time. Darwin and Wallace's theory of evolution via natural selection transformed biology and how we comprehend the historical origins of our species. Franklin, Crick, and Watson's discovery of the double helix structure of DNA radically redefined genetics and how we conceive the way genetic information of living organisms is stored, copied, and passed along. These scientists fundamentally changed the way we view the world, but they did not carry out an experiment to make these path-breaking discoveries. In fact, hundreds of major scientific discoveries did not use “the scientific method”, as defined in science dictionaries as the combined process of “the collection of data through observation and experiment, and the formulation and testing of hypotheses” (1). In other words, it is “The process of observing, asking questions, and seeking answers through tests and experiments” (2, cf. 3). Many recent science textbooks also present the scientific method as a sequence of steps or a process of observing, experimenting, and testing hypotheses, as shown in systematic studies of university-level science textbooks across science (4–7). The common scientific method is thus embedded in science dictionaries and textbooks (4–7). A study of major science institutions like the National Science Foundation and National Institutes of Health also found that they primarily endorse this scientific method focused on hypothesis testing, and generally not other exploratory research methods that do not test a predefined hypothesis (8). Researchers have not however yet used large representative data to assess the extent to which the scientific method is actually applied in science or they investigate it at an abstract level (9, 10). In general, this universal method is commonly viewed as a unifying method of science and can be traced back at least to Francis Bacon's theory of scientific methodology in 1620 which popularized the concept (11). This seminal book in many ways has laid the foundation of philosophy of science and fundamentally influenced generations of scientists and the common conception of how science is conducted, which remains widespread and institutionalized today (4–8, cf.^12^).

However, before hypothesizing about science, what its general method is and how it should be conducted, we need to first assess the evidence on how science is actually conducted in practice. Assessing science's major discoveries across scientific fields and time provides a new systematic way to do so and enables us to evaluate how this universal concept of scientific methodology holds up. Science's major discoveries are defined here as all 533 Nobel Prize–winning discoveries in science (from the first year of the prize in 1901 to 2022) (13) and all other major discoveries that were made prior to or did not receive a Nobel Prize; these are derived from all science textbooks (a total of seven) that provide a top 100 list of the greatest scientists and their discoveries and that span across scientific fields and history (14–20) (with textbooks specific to a field or time period not included). After excluding duplicate cases within the seven textbooks, 228 other major discoveries remained. A total of 761 major discoveries, which have driven humankind's knowledge, have thus been included in the study. The main source for compiling the data in this study is the main publication of the discovery that indicates the methods used to make the breakthrough (in the case of discoveries earning a Nobel Prize, the prize-winning papers) (13). For further description of the data, see figure captions (for overall greater details on the data, see the companion study that outlines the features and characteristics of science's major discoverers) (21).

Examining science's major discoveries, we find that the common scientific method (the combined use of observation, experimentation, and hypothesis testing) is applied in making 71% of all discoveries; and the share is 75% for all discoveries in contemporary science, defined as all Nobel Prize and major non-Nobel Prize discoveries since 1900. Among all major scientific discoveries, we find that 94% have required using observation, 81% testing a hypothesis, and 75% experimentation (Fig. 1)—with some hypotheses tested using experimental research designs and others using only observation. Science thus does not always fit the textbook definition.

**Fig. 1.**
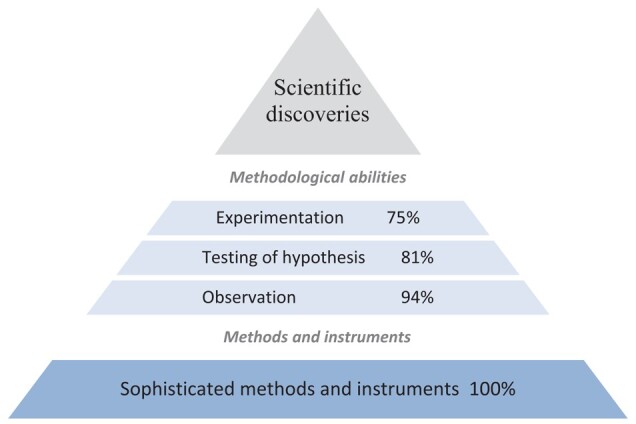
*Methods of science pyramid*: share of each methodological approach used for making discoveries. Data reflect all 761 major discoveries.

Comparison across fields provides evidence that the common scientific method was not applied in making about half of all Nobel Prize discoveries in astronomy, economics and social sciences, and a quarter of such discoveries in physics, as highlighted in Fig. 2b. Some discoveries are thus non-experimental and more theoretical in nature, while others are made in an exploratory way, without explicitly formulating and testing a preestablished hypothesis. Importantly, the common scientific method does not take into account that all Nobel Prize discoveries across fields require applying sophisticated methods (such as statistics and randomization techniques) or instruments (such as centrifuges and computers)—Fig. 2b.

**Fig. 2.**
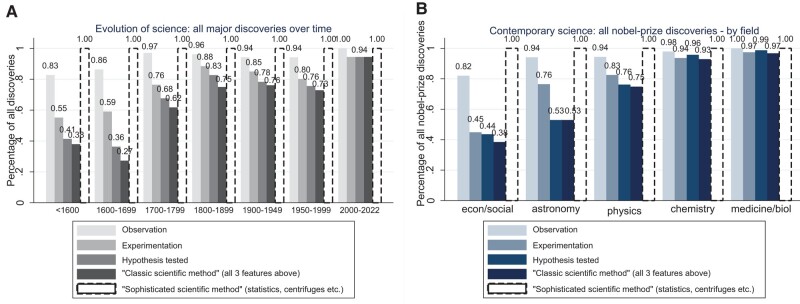
Share of discoveries made using the classic and the sophisticated scientific method, across time and fields. Data reflect all 761 major discoveries (including all Nobel Prize discoveries) (a), and all 533 Nobel Prize discoveries (b). Each of these discovery-making publications are classified as using observation if the study describes collecting observational data (using eyesight) (bar 1 in the figure), as using experimentation if the study conducted an experiment (bar 2), and as testing a hypothesis if the study formulated and assessed a proposed explanation (rather than conducted exploratory research) (bar 3). The publication is classified as using the *classic scientific method* if the study applied the three features (bar 4). In contrast, the publication is classified as using the *sophisticated scientific method* if the study applied a complex scientific method or instrument (bar 5), as defined below. The 10 most commonly used scientific methods and instruments—among all Nobel Prize discoveries—include statistical/mathematical methods, spectrometers, X-ray methods, chromatography, centrifuges, electrophoresis, lasers, (electron) microscopes, particle accelerator, and particle detector. Analysis expanding the data in (b) to include, in addition, the other major discoveries that did not earn a Nobel Prize but were made within the same time period (633 discoveries in total) illustrates comparable results (except for astronomy) and serves as a robustness check, with for example the share of discoveries made applying “the classic scientific method” at 40, 35, 75, 93, and 89% across these five fields, respectively.

When we systematically assess all major discoveries, what is the common method of science that we use to be able to do science and make discoveries? We find that one general feature of scientific methodology is applied in making science's major discoveries: the use of sophisticated methods or instruments. These are defined here as scientific methods and instruments that extend our cognitive and sensory abilities—such as statistical methods, lasers, and chromatography methods. They are external resources (material artifacts) that can be shared and used by others—whereas observing, hypothesizing, and experimenting are, in contrast, largely internal (cognitive) abilities that are not material (Fig. 2). Applying sophisticated methods or instruments is thus a necessary condition for discovery in contemporary science. We find that a number of sophisticated methods and instruments have each been used in making at least 10% of all major discoveries, such as centrifuges, X-ray diffraction, and spectrometers—and statistical methods for example have been used in making 62% of all discoveries. Without such scientific tools, discovery and scientific progress is not possible.

In fact, this sophisticated scientific method is actually more unique to science, as the most common scientific methods and instruments—such as particle accelerators, electrophoresis methods, and X-ray diffraction—are largely only used in science. In contrast, we also often make observations, test hypotheses, and experiment in business, industry, public policy, and everyday life and they are thus not just prototypical or distinctive of science. Recognizing the vast importance of such complex methods and instruments adds an essential element to understanding science and especially how science has evolved from its early origins in directly observing, hypothesizing and experimenting to now only being able to do so by using such complex tools. The *classic scientific method* dominated how science was done for much of history (especially when early scholars like Bacon described it) (11) but now *sophisticated scientific methods* dominate contemporary science by enabling us to observe, experiment, and test hypotheses in much more diverse, complex, and efficient ways. *Just as science has evolved, so should the classic scientific method—which is construed in such general terms that it would be better described as a basic method of reasoning used for human activities* (non-scientific and scientific).

While features of science such as observation, experimentation, and hypothesis testing are commonly used in science and making discoveries, they are thus not universal. An experimental research design was not carried out when Einstein developed the law of the photoelectric effect in 1905 or when Franklin, Crick, and Watson discovered the double helix structure of DNA in 1953 using observational images developed by Franklin. Direct observation was not made when for example Penrose developed the mathematical proof for black holes in 1965 or when Prigogine developed the theory of dissipative structures in thermodynamics in 1969. A hypothesis was not directly tested when Jerne developed the natural-selection theory of antibody formation in 1955 or when Peebles developed the theoretical framework of physical cosmology in 1965. These scientists all earned a Nobel Prize for these discoveries, but they did not directly apply or generally could not apply the “scientific method” to make their discovery. The common scientific method captures much of scientific practice but not all domains. If we were to abide by the common definition of the scientific method, Copernicus (22), Darwin (23), Einstein (24), Franklin, Crick, and Watson, and many others would not be viewed as having applied it as they did not directly carry out experiments to make their seminal breakthroughs. These scientists have however become iconic figures of science.

In general, scientific methods—like scientists—come in many sizes, shapes, and levels of sophistication. We use many methods to conduct science across fields: combining mathematics with measurement instruments, statistics with experimentation, X-ray diffraction, spectrometers, and particle detectors using systematic observation, and hundreds of other combinations. We may think of the diverse methods needed in immunology, oceanography, neuroscience, and astrophysics, or chemistry, agronomy, and behavioral economics. We cannot do science without our sophisticated methods and instruments which make it possible, for most phenomena in science, to observe, experiment, and test hypotheses and especially do exploratory research in the first place—and also to do so in new and innovative ways (Table 1). The *sophisticated scientific method* integrates the use of observation, experimentation, and hypothesis testing into our central methods and instruments (Fig. 3). Replicability, a central feature of science, is also tied to particular sophisticated methods, such as statistical methods and X-ray devices. Different researchers applying sophisticated methods ensures that studies, theories, and discoveries are replicable (while observation, experimentation, and hypothesis testing are too general to do so and are subject to each researcher applying them differently and thus more susceptible to researcher bias). Sophisticated methods make research more accurate and reliable and enable us to evaluate the quality of research.

**Fig. 3.**
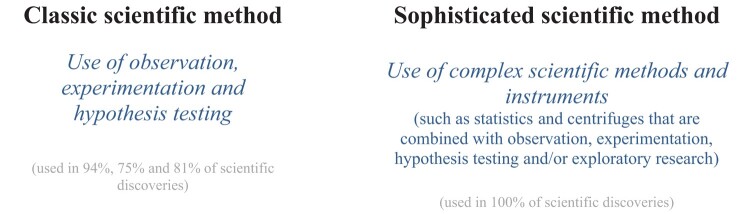


**Table 1.** Main types of methodological approaches used in science and making discoveries.

	Theoretical sciences	Empirical sciences	
		Experimental sciences	Observational sciences
Methodological approach used to make discoveries:	No observation or experimentation, but only a method or instrument	Observation, experimentation, and a method or instrument	Observation, and a method or instrument
Share of all major discoveries over history	6%	75%	94%
Share of all Nobel Prize discoveries only	5%	82%	95%
Examples of discoveries	Mathematical proof for black holes	Immunity factors of blood serum	Pulsars
	Feynman diagrams	Econometrics	Structure of DNA molecule
	Nash equilibrium	Energy production in stars	Accelerating expansion of universe

Data reflect all 761 major discoveries (including all Nobel Prize discoveries) (first row of data), and all 533 Nobel Prize discoveries (second row of data). Applying observation and a complex method or instrument, together, is decisive in producing nearly all major discoveries at 94%, illustrating the central importance of empirical sciences in driving discovery and science.

Overall, with the classic scientific method, we would not be able to label many major scientific discoveries as scientific, though they have vastly impacted science and our lives. The concept of the common scientific method, as a golden principle connecting the scientific community together, can be misunderstood as being universal. It is an idealization, embedded in university science textbooks (4–7), science dictionaries (1–3), and several major science institutions (8), that can be confusing for students and less-experienced researchers when learning about science and scientific discoveries and realizing it does not always apply. We do science and make breakthroughs using our diverse and complex methodological toolbox. *We can best view the method of science as the use of our sophisticated methodological toolbox*. The *classic scientific method* needs to be integrated into and redefined as the *sophisticated scientific method* that better reflects actual scientific practice:

Scientific methodology is defined as the use of sophisticated scientific methods or instruments (such as mathematics, particle accelerators, and chromatography methods), which are systematic techniques and tools that extend our cognitive and sensory abilities, are generalizable and enable better observing, hypothesis-testing, problem-solving, and experimenting and thus acquiring knowledge about the world.

A generalizable method or instrument means that it is applicable in different contexts to do science. This definition can provide a more accurate understanding of the nature of scientific methodology. It also directs our attention to refining and expanding our sophisticated methodological toolbox that is what enables us to drive science and push the scientific frontier. Other features of science’s major discoveries are outlined in a series of forthcoming papers and forthcoming book *The Motor of Scientific Discovery*. Ultimately, the best path to discovery is not the classic scientific method but the sophisticated scientific method.

## Supplementary Material

pgae112_Supplementary_Data

## Data Availability

Data used for the analysis are available in Dataset S1 and from the sources outlined in the text.
