# Correction to: Redefining the scientific method: as the use of sophisticated scientific methods that extend our mind

**DOI:** 10.1093/pnasnexus/pgae406

**Published:** 2024-10-30

This is a correction to: Alexander Krauss, Redefining the scientific method: as the use of sophisticated scientific methods that extend our mind, *PNAS Nexus*, Volume 3, Issue 4, April 2024, pgae112, https://doi.org/10.1093/pnasnexus/pgae112

Dataset S1 has been added as supplementary data and the data availability statement has been updated to read, “Data used for the analysis are available in Dataset S1 and from the sources outlined in the text.”

